# Effect of surface properties of silica nanoparticles on their cytotoxicity and cellular distribution in murine macrophages

**DOI:** 10.1186/1556-276X-6-93

**Published:** 2011-01-18

**Authors:** Hiromi Nabeshi, Tomoaki Yoshikawa, Akihiro Arimori, Tokuyuki Yoshida, Saeko Tochigi, Toshiro Hirai, Takanori Akase, Kazuya Nagano, Yasuhiro Abe, Haruhiko Kamada, Shin-ichi Tsunoda, Norio Itoh, Yasuo Yoshioka, Yasuo Tsutsumi

**Affiliations:** 1 Department of Toxicology and Safety Science, Graduate School of Pharmaceutical Sciences, Osaka University, 1-6 Yamadaoka, Suita, Osaka 565-0871, Japan; 2 Laboratory of Biopharmaceutical Research (Pharmaceutical Proteomics), National Institute of Biomedical Innovation, 7-6-8 Saito-Asagi, Ibaraki, Osaka 567-0085, Japan; 3 The Center for Advanced Medical Engineering and Informatics, Osaka University, 1-6 Yamadaoka, Suita, Osaka 565-0871, Japan; 4 Department of Biomedical Innovation, Graduate School of Pharmaceutical Sciences, Osaka University, 7-6-8, Saito-Asagi, Ibaraki, Osaka 567-0085, Japan

## Abstract

Surface properties are often hypothesized to be important factors in the development of safer forms of nanomaterials (NMs). However, the results obtained from studying the cellular responses to NMs are often contradictory. Hence, the aim of this study was to investigate the relationship between the surface properties of silica nanoparticles and their cytotoxicity against a murine macrophage cell line (RAW264.7). The surface of the silica nanoparticles was either unmodified (nSP70) or modified with amine (nSP70-N) or carboxyl groups (nSP70-C). First, the properties of the silica nanoparticles were characterized. RAW264.7 cells were then exposed to nSP70, nSP70-N, or nSP70-C, and any cytotoxic effects were monitored by analyzing DNA synthesis. The results of this study show that nSP70-N and nSP70-C have a smaller effect on DNA synthesis activity by comparison to unmodified nSP70. Analysis of the intracellular localization of the silica nanoparticles revealed that nSP70 had penetrated into the nucleus, whereas nSP70-N and nSP70-C showed no nuclear localization. These results suggest that intracellular localization is a critical factor underlying the cytotoxicity of these silica nanoparticles. Thus, the surface properties of silica nanoparticles play an important role in determining their safety. Our results suggest that optimization of the surface characteristics of silica nanoparticles will contribute to the development of safer forms of NMs.

## Introduction

Recently, a range of nanomaterials (NMs) have been designed and used in a number of different industrial applications, such as medicine, cosmetics, and foods. The application of NMs is driven by the belief that they will offer improved performance and deliver new functionalities, including improved thermal and electrical conductivity, harder and stronger materials, improved catalytic activity, and advanced optical properties. For example, current estimates indicate that the global market for cosmetics using NMs will grow by 16.6% per year, reaching US$ 155.8 million in 2012 [1]. Hence, human exposure to NMs is already occurring and will inevitably increase in the future.

A NM is defined as a substance that has at least one dimension of <100 nm in size. NMs can assume many different forms, such as tubes, rods, wires, spheres, or particles. However, their small size can also be problematic in terms of eliciting a toxicological effect. For example, exposure of cells or animals to carbon nanotubes, titanium dioxide particles, or silver nanoparticles can induce cytotoxicity and inflammation [2-14]. We have previously shown that silica nanoparticles display a different intracellular localization compared with submicron- and micro-sized silica particles, and induce a greater cytotoxic response [15]. However, analyses of the toxicological responses to NMs are often inconsistent. Given the uncertainty concerning the safety of NMs, it is critically important to analyze their potential toxicological hazards and devise means of minimizing the impact of exposure to such substances. These studies will assist in driving forward the nanotechnology industry in the longer term by helping the researchers to protect both individuals and the environment from potentially damaging materials.

Some recent articles have focused on the possible influence of surface charge in terms of the cellular uptake and/or cytotoxicity of nanoparticles [16-19]. Mayer et al. [19] reported the activation of the complement system and increased hemolysis in blood samples after exposure to positively charged polystyrene nanoparticles. Some recent studies suggest that cationic nanoparticles elicit a greater cytotoxicity compared with anionic nanoparticles [20-22]. Taken together, these studies indicate that the surface property of nanoparticles is an important factor when developing safer forms of NMs. However, studies of cellular responses to NMs often give conflicting results. The aim of this study was to investigate the cytotoxicity caused by exposure of a murine macrophage cell line (RAW264.7) to silica nanoparticles whose surface was either unmodified (nSP70) or modified with amine (nSP70-N) or carboxyl groups (nSP70-C). The intracellular localization of the different nanoparticles was also examined.

## Experimental procedures

### Silica particles

Fluorescent (red-F or green-F)-labeled silica particles with surfaces that were either unmodified or modified with amine or carboxyl groups (Micromod Partikeltechnologie GmbH, Rostock, Germany; designated nSP70, nSP70-N, and nSP70-C, respectively) were used in this study. The silica particles, which had a diameter of 70 nm, were prepared as a suspension (25 mg/ml) and sonicated for 5 min and then vortexed for 1 min immediately prior to conducting each experiment.

### Physicochemical examination of the nanosilica preparations

Nanosilicas were diluted to 0.25 mg/ml with water, and the average particle size and zeta potential were measured using a Zetasizer Nano-ZS (Malvern Instruments Ltd., Malvern, UK). The mean size and the size distribution of silica particles were measured by dynamic light scattering. The zeta potential was measured by laser Doppler electrophoresis.

### Cell culture

The mouse macrophage cell line, RAW 264.7, was obtained from the American Type Culture Collection. RAW 264.7 cells were cultured in Dulbecco's Modified Eagle Medium supplemented with 10% heat-inactivated FCS, 1% Antibiotic-Antimycotic Mix stock solution (GIBCO, CA, USA). All cultures were incubated at 37°C in a humidified atmosphere with 5% CO_2_.

### ^3^H-Thymidine incorporation assay

The proliferation of nanosilica-treated RAW 264.7 cells and untreated cells was measured using a ^3^H-thymidine incorporation assay. 10^4 ^cells were cultured with varying concentrations of nanosilica diluted with medium for 18 h at 37°C, and ^3^H-thymidine (1 μCi/well) was then added into each well. After a further 6 h, cells were harvested and lysed on glass fiber filter plates using a Cell harvester (Perkin-Elmer, Wellesley, MA, USA). The filter plates were then dried and counted by standard liquid scintillation counting techniques in a TopCounter (Perkin-Elmer).

### Confocal scanning laser microscopy analysis of the macrophage cell line

RAW 264.7 cells were cultured with nSP70, nSP70-N, and nSP70-C (100 μg/ml) for 3 h on chamber slides, then fixed at room temperature in 4% paraformaldehyde and washed three times in 0.1 M phosphate buffer (pH 7.4). Cells were then filled with mounting medium containing 4',6-diamino-2-phenylindole (DAPI) (Vector Laboratories, Burlingame, CA, USA). A glass cover slip was then placed on the slide and fixed with glue. The mounted slides were examined under a confocal scanning laser microscope (Leica Microsystems, Mannheim, Germany).

## Results and discussion

First, the authors assessed the mean particle size and surface charge of 70 nm silica particles in water whose surface was unmodified (nSP70) or chemically modified with amine (nSP70-N) or carboxyl groups (nSP70-C). The results are summarized in Table 1. Mean particle sizes of nSP70, nSP70-N, and nSP70-C as measured by dynamic light scattering method were 64.2 ± 0.6, 72.7 ± 1.3, and 76.2 ± 1.6 nm, respectively. These experimentally determined particle sizes were almost equal to the primary diameter sizes (70 nm). Surface charges (zeta potential) of nSP70-N and nSP70-C were, respectively, higher and lower compared to those of nSP70.

**Table 1:** Average particle size and zeta potential of unmodified and modified nanosilica

	nSP70	nSP70-N	nSP70-C
Modification substance	-	NH_2_	COOH
Mean particle size in water (nm)	64.2 ± 0.6	72.7 ± 1.3	76.2 ± 1.6
Mean zeta potential (mV)	-42.1 ± 0.6	-29.8 ± 0.5	-72.0 ± 1.9

Each value represents the mean ± SD

**Figure 1:**
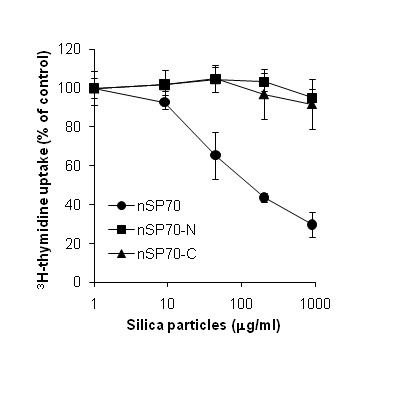
**Effect of unmodified and modified nanosilica on cell proliferation**. The proliferation of RAW 264.7 cells after incubation with nSP70 (circle), nSP70-N (square), or nSP70-C (triangle) for 24 h was evaluated using the ^3^H-thymidine incorporation assay. The percentage increase in cell proliferation was calculated relative to the negative control. Data are presented as means ± SD.

Cytotoxicity of the three nanosilicas was tested by monitoring the incorporation of ^3^H-thymidine into RAW 264.7 cells. nSP70 showed the highest cytotoxicity (EC50 value = 121.5 μg/ml), while nSP70-N and nSP70-C failed to display any detectable cytotoxicity up to concentrations of 1000 μg/ml (Figure 1). These results demonstrate that the cytotoxic effect of nSP70 is reduced by surface modification. Some reports indicated that the exposure of cells to silica nanoparticles leads to membrane damage, caspase activation, and cell death via apoptosis. The precise trigger for these silica nanoparticle-induced cellular effects is uncertain. One study concluded that lysosomal destabilization was the initiation factor [23], whereas other investigations suggest that mitochondrial membrane damage is the critical event [24,25]. However, it is possible that multiple factors (i.e., membrane damage, caspase activation, lysosomal destabilization, and mitochondrial membrane damage) are involved in nSP70-mediated cytotoxicity. Unfortunately, it is difficult to establish a comprehensive mechanism of nSP70 cytotoxicity based on previous observations, which have been rather inconsistent.

**Figure 2:**
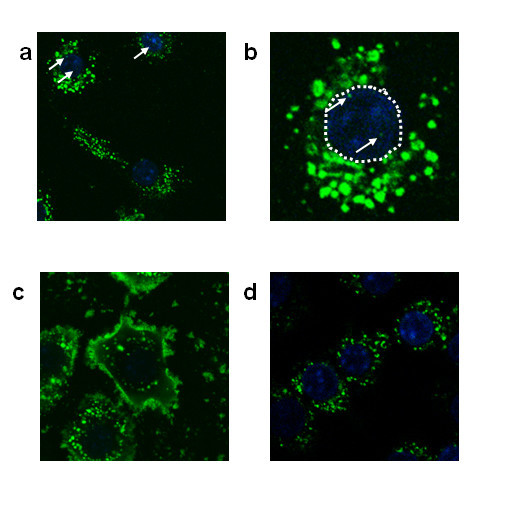
**Localization of unmodified and modified nanosilica in RAW 264.7 cells**. RAW 264.7 cells were treated for 3 h with 100 μg/ml of fluorescent (green-F)-labeled nSP70 **(a, b)**, nSP70-N **(c)**, and nSP70-C **(d) **(green). The nucleus was counterstained using DAPI (blue). The original magnification of these photographs was × 63 (a) and × 100 (c, d). (b) is a magnified image of a portion of photograph (a).

Some reports indicate that the cellular uptake and trafficking of nanoparticles is involved in cellular signaling, which then leads to cytotoxicity. However, the relationship between the surface properties of nanoparticles and cellular uptake/trafficking is poorly understood. To establish why a different biological effect was induced by surface modification, the intracellular localization of fluorescent-labeled nanosilicas was investigated. RAW 264.7 cells treated with 100 μg/ml of nSP70, nSP70-N, and nSP70-C were observed by confocal laser scanning microscopy. After 3-h incubation, all nanosilicas were found to be localized in the cytoplasm as punctate fluorescent dots regardless of surface modification (Figure 2). Interestingly, distinctive distributions were observed in individual cases. For the nSP70-treated cells, punctate fluorescence was observed in both the cytoplasm and nucleus. However, nSP70-N appeared to adsorb to the plasma membrane because bright fluorescence was observed along the outline of the cell. In the nSP70-C-treated group, only intracellular punctate fluorescence was observed, suggesting that these particles were efficiently incorporated into the cells. Unfortunately, detailed intracellular localization of the silica nanoparticles was not apparent from these images. Nonetheless, differences of intracellular localization of nanosilica might have an effect on cytotoxicity. It is reported that the entry of nanosilica into the nucleus induces dysfunction of the nucleus and genotoxicity via aggregation of intranuclear protein or inhibition of RNA transcription [26]. The said report [26] together with the results of this study suggests that silica nanoparticles enter the nucleus and induce inhibition of cell proliferation. Hence, the nSP70-mediated cytotoxic effect may be related to nuclear localization.

Investigating the cellular uptake/trafficking of individual nanosilicas is important for the development of safer forms of NMs. It is known that surface chemistry of nanoparticles, such as charge and the kind of modification group, affects their interaction with biological molecules [27]. For example, nanoparticles can induce different cellular responses by binding to proteins in the blood [28,29]. Bound proteins determine particle uptake by various cells and influence how nanoparticles interact with other blood components [30-32]. These findings suggest that surface modification alters the interaction between nanosilica and surrounding molecules, such as serum proteins, thereby altering the route of uptake into the cells. Mammalian cells ingest particulate matter by several routes, such as phagocytosis, macropinocytosis, clathrin-mediated, caveolin-mediated, and clathrin/caveolin independent endocytosis [33-35]. Each route involves a unique set of receptors and acts on particular types of particles. The authors anticipate that surface modification of silica nanoparticles will influence their interaction with bloodborne macromolecules. Thus, nanoparticles decorated with different macromolecules will have different intracellular distributions. The authors are currently investigating the effects of nSP70, nSP70-C, and nSP70-N on cytotoxicity, protein adsorption, cellular uptake, ROS generation, lysosomal stability, mitochondrial activity, activation of caspase 3 and 7, and mode of cell death (apoptosis versus necrosis).

In this study, the authors demonstrate that surface modification of nSP70 with amine or carboxyl groups alters the intracellular distribution of the nanoparticles and has an effect on cell proliferation. The authors believe that the identification of individual uptake machinery will shed light on the safety of nanosilicas, which are already commercially available in the form of medicines, cosmetics, and foods. Furthermore, it is hoped that analysis of the relationship between surface physicochemical properties and cellular response/distribution will help researchers in the development of safer forms of NMs. A safety-prediction as well as safety-evaluation approach of NMs is an essential prerequisite for maintaining the well-being of the general public.

## Abbreviations

DAPI: 4',6-diamino-2-phenylindole; NMs: nanomaterials.

## Competing interests

The authors declare that they have no competing interests.

## Authors' contributions

HN and T. Yoshikawa designed the study; HN, AA, T. Yoshida, S. Tochigi, TH and TA performed experiments; HN and T. Yoshikawa collected and analysed data; HN and T. Yoshikawa wrote the manuscript; KN, YA, HK, S. Tsunoda, NI and YY gave technical support and conceptual advice. YT supervised the all of projects. All authors discussed the results and commented on the manuscript.
